# Genotyping and molecular investigation of plasmid-mediated carbapenem resistant clinical *Klebsiella pneumoniae* isolates in Egypt

**DOI:** 10.3934/microbiol.2023014

**Published:** 2023-03-23

**Authors:** Kholoud Baraka, Rania Abozahra, Marwa Mohammed Haggag, Sarah M Abdelhamid

**Affiliations:** 1 Microbiology and Immunology Department, Faculty of Pharmacy, Damanhour University, El Behira, Egypt; 2 Microbiology and Immunology Department, Faculty of Pharmacy, Sinai University, Arish Campus, Sinai, Egypt

**Keywords:** *Klebsiella*, Egypt, carbapenem, genotyping, plasmid

## Abstract

*Klebsiella pneumoniae* is a multidrug-resistant nosocomial pathogen. Carbapenem resistance is mediated mainly by enzymes carried on transmissible plasmids causing their dissemination among other members of *Enterobacteriaceae*. This study aimed to molecularly detect carbapenem resistance genes in *K. pneumoniae* clinical isolates, genotype them using ERIC-PCR, and investigate plasmid transformation of resistant genes by using ERIC-PCR and sequencing.

**Methods:**

Antimicrobial resistance of sixty carbapenem-resistant *K. pneumoniae* strains was evaluated by using the disc diffusion method. Five carbapenemases' genes were amplified by conventional PCR. Genotyping was performed using ERIC-PCR. Gene transformation was performed for the five genes to sensitive isolates. Wild and transformed isolates were genetically investigated using ERIC-PCR and sequencing.

**Results:**

Carbapenem resistance in our isolates was associated with high resistance to all tested antibiotics. The 60 *K. pneumoniae* isolates were divided into 6 resistor types. The prevalence of *KPC, IMP, VIM, NDM*, and *OXA-48* genes were 17%, 63%, 93%, 85% and 100%, respectively. Dendrogram analysis showed 57 distinct patterns, arranged in three clusters. The five genes were transformed successfully into sensitive isolates. ERIC profiles of wild and transformed isolates showed cluster A contained all the wild isolates, and cluster B contained all transformed isolates. Genetic sequences of the 5 genes reflected high genetic similarity with the GenBank reference genes before plasmid transformation; however, a distinguishable decrease of genetic similarity was observed after transformation.

**Conclusion:**

Plasmid-mediated carbapenem resistance in *K. pneumoniae* and its dissemination among different strains is a real threat to public health.

## 1. Introduction

*Klebsiella pneumoniae* (*K. pneumoniae*) is a Gram-negative rod-shaped bacterium that is highly virulent and is frequently associated with antibiotic resistance. In humans, *K. pneumoniae* can cause serious diseases including pneumonia, liver abscesses, urinary tract infections, and life-threatening septicemia [1]–[3]. In Egypt, nosocomial infections are primarily caused by *K. pneumoniae* [4]. It functions as a pool for transmissible plasmids carrying different antibiotic-resistance genes. This makes *K. pneumoniae*-resistant strains an alarming threat in hospitals and healthcare institutions, as these resistance genes can spread to other members of *Enterobacteriaceae*. In addition, plasmids carrying resistant genes can travel with patients to disseminate the resistance to different geographic areas [5].

Carbapenem antibiotics were used as a last resort to treat multidrug-resistant *K. pneumoniae* strains until carbapenem-resistant strains emerged all over the world [6]–[8]. In addition, the CDC has declared carbapenem-resistant *Enterobacteriaceae* a public health emergency. In the United States in 2019 [9], carbapenem resistance in *K. pneumoniae* was mediated by one of three mechanisms. The first mechanism is the production of carbapenemases, the second is the hyperproduction of AmpC β-lactamase accompanied by an outer membrane porin mutation, and the third is the extended-spectrum β-lactamase (ESBL) production with a porin mutation or drug efflux. The production of carbapenemases is the most commonly reported resistance mechanism [10].

In *K. pneumoniae* the main genes responsible for carbapenem resistance are *K. pneumoniae* carbapenemases (*blaKPC*), New Delhi metallo-β-lactamases (*blaNDM*), Verona integron-encoded metallo-β-lactamases (*blaVIM*), imipenem metallo-β-lactamases (*blaIMP*) and oxacillinase-48 (*OXA*-48) carbapenemases. *K. pneumoniae* carbapenemases (*KPC*) belong to the *Ambler* Class A carbapenemases. They have high activity against carbapenems as well as a variety of β-lactam antibiotics such as penicillins, cephalosporins, and monobactams. Moreover, they are inhibited by the *in vitro* activity of β-lactamase inhibitors such as clavulanic acid and sulbactam. Furthermore, the *KPC* genes are located on plasmids carrying genes for resistance to other antibiotic classes such as fluoroquinolones and aminoglycosides. New Delhi metallo-β-lactamases (*NDM*), Verona integron-encoded metallo-β-lactamases (*VIM*), and imipenem metallo-β-lactamases (*IMP*) belong to class B carbapenemases. They have a zinc group on their active site so they are inhibited by metal-chelating agents such as ethylenediaminetetraacetic acid (EDTA). They confer resistance to penicillins, cephalosporins, carbapenems, and β-lactamase inhibitors. OXA-48-like carbapenemases belonging to the *Ambler* Class D carbapenemases have weak activity against carbapenems and are not inhibited by EDTA or clavulanic acid [11],[12]. These carbapenemases are plasmid-mediated and can be easily disseminated in the community as well as in hospital wards conferring a great impact on public health [13]. In Egypt, many studies showed a high level of carbapenem resistance among hospital *K. pneumoniae* isolates [14],[15].

Molecular typing techniques are valuable tools for revealing the genetic relationships in nosocomial infection outbreaks, identifying the likely source of infection, and helping in the management and treatment of infections caused by multi-drug resistant pathogens. Enterobacterial repetitive intergenic consensus-polymerase chain reaction (ERIC-PCR) is a molecular technique that is used to gauge the genetic diversity among members of the *Enterobacteriaceae* family. The 126 bp length ERIC sequences are noncoding, and conserved regions. The ERIC approach can be used to assess genetic variations between bacterial isolates. ERIC sequences can be found in bacteria in a variety of locations and quantities. The ERIC-PCR is a quick, accurate, and reliable method for examining the genetic similarity of bacterial isolates [16]–[18]. This study aimed to investigate the prevalence of carbapenem resistance among clinical *K. pneumoniae* isolates and their genetic relatedness which constitute an important healthcare problem. Moreover, it aimed to detect carbapenem resistance genes abundant in *K. pneumoniae* strains isolated from Damanhour Medical National Institute and investigate their transferability to be capable of following up bacterial resistance and transmission in hospitals.

## 2. Materials and methods

### 2.1. Sample collection, isolation, and identification

One hundred different samples were collected from patients at Damanhour Medical National Institute, El-Behira, Egypt from March to May 2019. Urine, sputum, wound swabs, and blood samples were collected. For the detection of *K. pneumoniae*, samples were cultured on MacConkey agar plates. Gram staining was performed on lactose-fermenting mucoid colonies. Several biochemical tests, including oxidase, triple sugar iron agar, indole, methyl red, Voges Proskauer, citrate, and catalase, were carried out [19]. *K. pneumoniae* isolates were identified at the species level using the automated VITEK 2 system (Bio-Merieux, l'Etoile, France).

### 2.2. Antibiotic susceptibility testing

The antibiotic resistance was determined by using the standard disc diffusion technique according to Bauer *et al*. [20]. Fifteen commercially available antibiotic discs (Oxoid^®^-UK) representing four different antibiotic classes were used to assess resistance to ampicillin (AMP 10 µg) [Beta-lactams class], ampicillin/sulbactam (SAM 20 µg) [Beta-lactams class], piperacillin/tazobactam (TZP 110 µg) [Beta-lactams class], ertapenem (ETP 10 µg) [Beta-lactams class], cefazolin (CZ 30 µg) [Beta-lactams class], cefoxitin (FOX 30 µg) [Beta-lactams class], ceftazidime (CAZ 30 µg) [Beta-lactams class], ceftriaxone (CRO 30 µg) [Beta-lactams class], cefepime (FEP 30 µg) [Beta-lactams class], gentamycin (CN 10 µg) [Aminoglycosides class], amikacin (AKN 30 µg) [Aminoglycosides class], ciprofloxacin (CIP 5 µg) [Quinolones class], cotrimoxazole (SXT 25 µg) [Sulfonamides class], tobramycin (TOB 30 µg) [Aminoglycosides class], and levofloxacin (LVX 5 µg) [Quinolones class] (Oxoid® Ltd, England) according to CLSI 2020 guidelines [21]. Minimum inhibitory concentration (MIC) and ESBL tests were performed by VITEK 2 (BioMerieux, L'Etoile, France).

### 2.3. Total genomic DNA extraction

Pure colonies of carbapenem-resistant isolates cultured on Luria-Bertani (LB) broth were used for chromosomal DNA and plasmid DNA extraction using PureLink Genomic DNA Mini Kit (Thermofischer^®^.USA) and DNA-spin Plasmid Purification Kit (iNtRon Biotechnology^®^), respectively according to the manufacturer's protocol. A nanodrop 2000 spectrophotometer (Thermofischer^®^.USA) was used to detect the concentration of DNA. DNA and plasmid lysates were stored at –20 °C for further use.

### 2.4. Molecular detection of carbapenemases genes by conventional PCR technique

Plasmid DNA extracts were tested for five carbapenemases genes by using a thermal cycler (SimpliAmp Applied Biosystems, Thermofischer®.USA), and DreamTaq PCR Master Mix (Thermofisher^®^. USA). Five pairs of primers were used (Table 1) [22]. PCR amplicons were then resolved on 1.5% agarose gel stained with ethidium bromide, then visualized using ultraviolet illumination. The following cycling parameters were used: initial denaturation at 95 °C for 7 min; followed by 30 cycles of denaturation at 90 °C for 30 secs, annealing at 52 °C for 1 min, and extension at 65 °C for 8 min, and a final extension at 65 °C for 16 min. This step was done for all strains, and then for the wild and transformed strains.

**Table 1.** Primers used for amplifying the five carbapenemases genes^22^.

The gene	The primers' sequence	Amplicon size (bp)
*IMP*	*IMP*-F: GGAATAGAGTGGCTTAAYTCTC	233 bp
	*IMP*-R: GGTTTAAYAAAACAACCACC	
*VIM*	*VIM*-F: GATGGTGTTTGGTCGCATA	390 bp
	*VIM*-R: CGAATGCGCAGCACCAG	
*OXA*	*OXA*-F: GCGTGGTTAAGGATGAACAC	438 bp
	*OXA*-R: CATCAAGTTCAACCCAACCG	
*NDM*	*NDM*-F: GGTTTGGCGATCTGGTTTTC	621 bp
	*NDM*-R: CGGAATGGCTCATCACGATC	
*KPC*	*KPC*-F: CGTCTAGTTCTGCTGTCTTG	798 bp
	*KPC*-R: CTTGTCATCCTTGTTAGGCG	

### 2.5. Molecular genotyping of *K. pneumoniae* isolates by using ERIC-PCR

ERIC-PCR typing was performed for Inter-Simple Sequence Repeat (ISSR) by using DreamTaq PCR Master Mix (Thermofisher^®^. USA) and ERIC-primer-F: 5′-ATG TAA GCT CCT GGG GAT TCAC-3′ and ERIC-primer-R: 5′-AAG TAA GTG ACT GGG GTG AGC G-3′ (Thermofisher^®^, USA) [23] according to the following cyclic conditions: initial denaturation at 95 °C for 3 min, followed by 40 cycles of denaturation at 95 °C for 30 sec, annealing at 55 °C for 30 sec. and extension at 72 °C for 1 min, and a final extension at 72 °C for 10 min. PCR products were resolved on 1.5% agarose gel stained with ethidium bromide, then visualized using ultraviolet illumination. The TotalLab Quant Analysis software (Version 1.0., TotalLab Ltd. United Kingdom) was used for analysis. This step was done for all strains, and then for the wild and transformed strains.

### 2.6. Plasmid transformation

Eight carbapenem-sensitive isolates (4 *K. pneumoniae* and 4 *E. coli*) obtained from the same hospital were used as recipient cells for plasmid-carrying carbapenem resistance genes. Competent cell preparation and transformation were done as follows: Bacterial suspensions of the sensitive isolates in LB broth were prepared. Turbidity was adjusted to reach 5 × 10^7^ cells/mL then pellets were collected by centrifugation at 4 °C at 4000 rpm for 10 min. Pellets were suspended with 20 mL ice-cold 0.1M CaCl_2_ solution and incubated on ice for 30 min. Competent cells were obtained by centrifugation again at 4 °C at 4000 rpm for 10 min. and the supernatant layer was discarded. Competent cells were suspended in 5 mL ice-cold 0.1M CaCl_2_ with 15% glycerol and stored at –80 °C for transformation [24].

For plasmid transformation, 5 µL of plasmid extract was added to 50 µL of competent cell aliquots and incubated on ice for 30 min., transferred to a 42 °C water bath for 30 sec., then transferred immediately on ice for 2 min. One mL of warmed LB broth was added before incubation in a shaking incubator at 37 °C, 200 rpm for 1 hr.

### 2.7. Determination of imipenem MIC using the Agar dilution method

Serial dilutions of imipenem were prepared according to CLSI guidelines [21]. One mL of each concentration was added to 9 mL of Muller Hinton agar media and poured into sterile Petri dishes. Bacterial suspensions with turbidity equal to 0.5 McFarland were prepared and 10 µL of each suspension was dropped on agar plates. *E. coli* ATCC25922 was used as a reference strain. Plates were incubated at 37 °C for 24 hrs.

### 2.8. Sequencing

The ABI PRISM^®^ 3100 Genetic Analyzer (Micron-Corp. Korea) was used for sequencing of the 5 carbapenemases genes in wild and transformed isolates. A gel documentation system (Geldoc-it, UVP, England) was applied for data analysis by using Totallab analysis software (ww.totallab.com, Ver.1.0.1). Positive amplicons were eluted from agarose gel by using E.Z.N.A.^®^ Gel Extraction Kit (V-spin, Omega Bio-TEK ^®^, USA) according to the manufacturer's protocol. Aligned sequences were analyzed on the NCBI website (http://www.ncbi.nlm.nih.gov/webcite) using BLAST for identity confirmation. The Genetic distances and MultiAlignments were computed by the Pairwise Distance method using ClusteralW software analysis (www.ClusteralW.com).

### 2.9. Statistical analysis

Correlations between the antibiotic resistance profile of the *K. pneumoniae* isolates, PCR pattern of carbapenem-resistant genes, and ERIC genotypes were statistically determined by using the Chi-square test and Monte Carlo method by using the IBM SPSS software package version 20.0 (Armonk, NY: IBM Corp). Significance of the obtained results was adjusted at the 5% level.

## 3. Ethical approval

This study adhered to the accepted principles of ethical conduct according to the approval reference number (918PM9) given by the Research Ethics Committee of the Faculty of Pharmacy, Damanhour University. Before testing and molecular analysis of their materials, all the available samples and patient data were gathered with informed ethical consent.

## 4. Results

**Figure 1.**
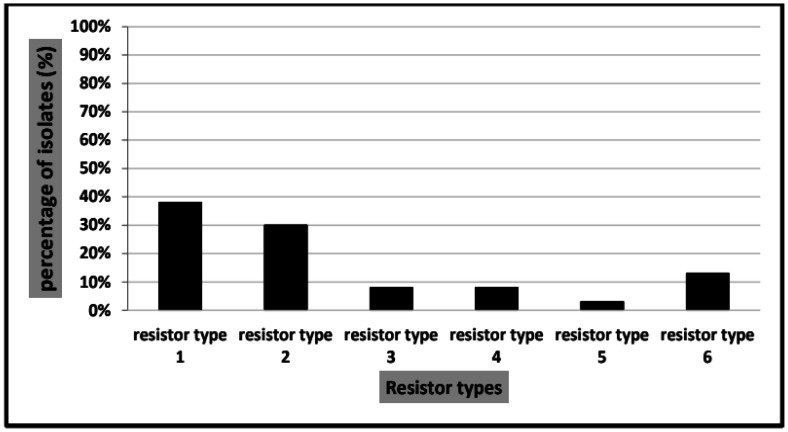
Prevalence of different resistor types among the 60 carbapenem resistant *K. pneumoniae* isolates.

Based on their appearance on MacConkey agar plates and morphological and biochemical characteristics, eighty (80%) of the 100 clinical isolates were initially identified as *Klebsiella spp*. Gram staining of mucoid lactose fermenting colonies on MacConkey agar plates revealed Gram negative rods. All isolates tested negative for oxidase, positive for catalase, negative for indole, negative for methyl red, positive for Voges Proskauer, and positive for citrate. All isolates produced acid butt and slant with gas and no H2S on triple sugar iron agar slants. Using the automated VITEK 2 system, 80 samples were identified as *K. pneumoniae*. Sixty (75%) of the 80 *K. pneumoniae* isolates were carbapenem resistant. Thirty-nine (65%) of the 60 *K. pneumoniae* isolates were obtained from sputum, 13 (21%) from urine, and 8 (14%) from wound swabs. Antibiotic sensitivity results showed that carbapenem resistance in our isolates was associated with high resistance to all tested antibiotics. All isolates were sensitive to all tested β-lactam antibiotics. Only 2 (3%) of the 60 carbapenem-resistant isolates were ESBL-positive. The 60 *K. pneumoniae* isolates were divided according to sensitivity pattern into 6 resistor types. 22 (38%) isolates were resistor type 1 (resistant to all tested antibiotics), 18 (30%) isolates were resistor type 2 (sensitive to 1 or more aminoglycosides), 5 (8%) isolates were resistor type 3 (sensitive to 1 or more aminoglycoside and trimethoprim-sulphamethoxazole), 5 (8%) isolates were resistor type 4 (sensitive to 1 or more fluoroquinolones), 2 (3%) isolates were resistor type 5 (sensitive to 1 or more aminoglycoside and quinolones), 8 (13%) isolates were resistor type 6 (sensitive to trimethoprim-sulphamethoxazole only) (Figure 1).

Molecular detection of carbapenem resistance genes using conventional PCR technique showed that all isolates contained at least two of the carbapenem resistance genes. All isolates were positive for the *OXA* gene (100%), 56 (93%) were positive for *VIM* gene, 51 (85%) were positive for *NDM* gene, 38 (63%) were positive for *IPM* gene, and 10 (17%) were positive for *KPC* gene. Seven (12%) isolates of the 60 *K. pneumoniae* isolates were positive for five resistance genes, 26 (43%) were positive for four genes, 22 (37%) were positive for 3 genes, and 5 (8%) were only positive for two genes. Resistant isolates were classified according to PCR results into nine groups as shown in Table 2.

**Table 2.** Distribution of 9 different PCR patterns among the 60 *K. pneumoniae* isolates.

PCR pattern	Resistance genes	No. of isolates (%)
1	IMP, VIM, OXA, NDM, KPC	7 (12%)
2	IMP, VIM, OXA, NDM	25(42%)
3	VIM, OXA, NDM, KPC	1 (2%)
4	VIM, OXA, NDM	16 (26%)
5	IMP, VIM, OXA	5 (8%)
6	OXA, NDM, KPC	1 (2%)
7	VIM, OXA	2 (3%)
8	OXA, NDM	2 (3%)
9	OXA+KPC	1 (2%)

**Figure 2.**
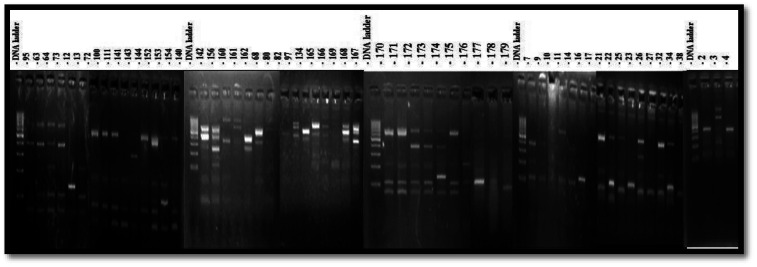
ERIC-PCR fingerprinting of carbapenem-resistant *K. pneumoniae* isolates showing different genotypes.

**Figure 3.**
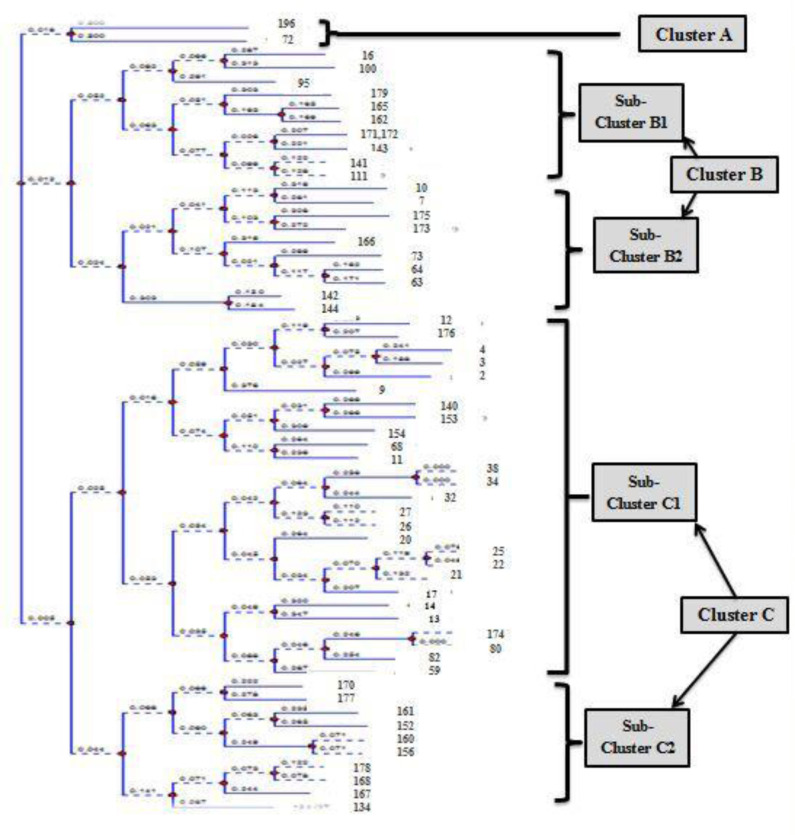
Dendrogram generated with Dice coefficient and the UPGMA clustering method, demonstrating the genetic similarity among *K. pneumoniae* isolates by Enterobacterial Repetitive Intergenic Consensus (ERIC) genotyping.

All isolates were genotyped by using ERIC-PCR and analyzed using TotalLab Quant Analysis software (Version 1.0., TotalLab Ltd. United Kingdom) to assess genetic similarity among our isolates. In this analysis the Dice method was used for comparison and the UPGMA method was used for clustering. Dendrogram analysis of *K. pneumoniae* isolates showed 59 distinct patterns, arranged in three clusters. Cluster A contained 2 isolates. Cluster B was divided into two sub-clusters: B1 (10 isolates) and B2 (10 isolates). Cluster C was divided into two sub-clusters: C1 (27 isolates) and C2 (10 isolates) (Figures 2 and 3). The resist type, PCR pattern, and ERIC profile of all isolates are illustrated in Table 3.

**Table 3.** The resistor type, PCR pattern, and ERIC profile of the 60 *K. pneumoniae* isolates.

Isolate code	Resist type	PCR pattern	ERIC profile
2	1	4	C1
3	2	9	C1
4	1	2	C1
7	1	2	B2
9	2	2	C1
10	1	2	B2
11	3	5	C1
12	2	2	C1
13	1	2	C1
14	2	1	C1
16	2	6	B1
17	2	5	C1
20	4	5	C1
21	1	4	C1
22	4	4	C1
25	1	4	C1
26	2	4	C1
27	1	4	C1
32	2	4	C1
34	1	4	C1
38	2	4	C1
59	2	4	C1
63	2	4	B2
64	1	2	B2
68	2	1	C1
72	2	4	A
73	6	2	B2
80	1	3	C1
82	2	1	C1
95	3	2	B1
100	1	5	B1
111	6	2	B1
134	1	2	C2
140	5	7	C1
141	5	5	B1
142	1	1	B2
143	2	2	B1
144	6	2	B2
152	1	4	C2
153	1	2	C1
154	1	4	C1
156	6	2	C2
160	6	2	C2
161	6	2	C2
162	6	2	B1
165	1	1	B1
166	1	7	B2
167	1	4	C2
168	1	1	C2
169	2	1	A
170	4	2	C2
171	2	2	B1
172	2	2	B1
173	1	2	B2
174	3	2	C1
175	4	4	B2
176	3	2	C1
177	3	2	C2
178	6	8	C2
179	4	8	B1

Plasmid transformation occurred successfully in the 8 carbapenem-sensitive *K. pneumoniae* and *E. coli* isolates. Both bacterial *spp*. succeeded to receive resistant plasmids from carbapenem resistant *K. pneumoniae* isolates. This was confirmed by the significant increase in MIC of imipenem after the transformation process using the agar dilution method (Table 4).

**Table 4.** Investigation of the plasmid transformation results by measuring MIC of imipenem before and after.

	Recipient organism	MIC of imipenem Before transformation (µg/mL)	MIC of imipenem After transformation (µg/mL)
1	*K. pneumoniae*	0.125	1
2	*K. pneumoniae*	0.125	4
3	*K. pneumoniae*	0.25	4
4	*E. coli*	0.5	2
5	*E. coli*	0.25	4
6	*E. coli*	0.25	32
7	*K. pneumoniae*	0.125	2
8	*E. coli*	0.125	8

**Figure 4.**
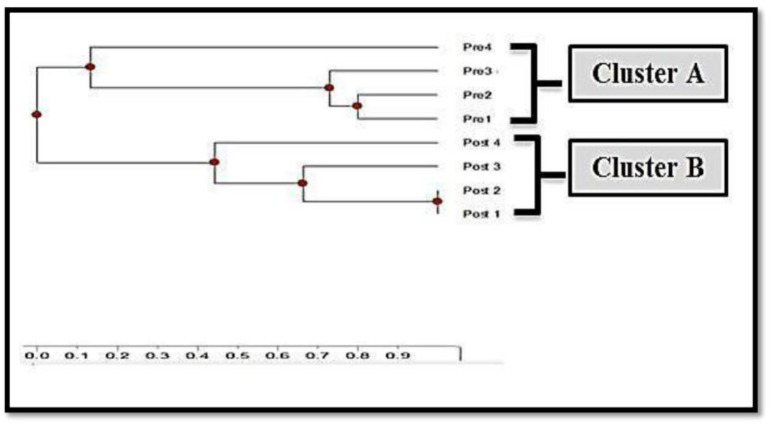
Dendrogram generated with Dice coefficient and the UPGMA clustering method, showing the genetic similarity among wild and transformed *K. pneumoniae* isolates by Enterobacterial Repetitive Intergenic Consensus (ERIC) genotyping.

Four wild carbapenem sensitive *K. pneumoniae* isolates were positive for the 5 genes. After plasmid transformation, the 5 genes were also detected in the 5 transformed carbapenem resistant *K. pneumoniae* isolates using conventional PCR. The 4 wild and 4 transformed *K. pneumoniae* isolates were genotyped by using ERIC-PCR, followed by analysis with TotalLab Quant Analysis software (Version 1.0., TotalLab Ltd. United Kingdom) to evaluate genetic similarity among our isolates. Dendrogram analysis showed 7 distinct patterns, arranged in two clusters. Cluster A contained all the wild (Pre-transformation) isolates, and Cluster B contained all the transformed (Post-transformation) isolates (Figure 4).

PCR amplicons were eluted, sequenced, aligned, and compared with reference genes. Generally, gene sequences of each wild isolate were more similar to the sequence of reference genes of the GenBank than its genes' sequences after transformation. Before plasmid transformation, genetic sequences of the 5 genes reflected high genetic similarity with the GenBank reference genes as follows: *VIM* (99.23%), *IMP* (99.12%), *OXA* (99.39%), *NDM* (100%), and *KPC* (99.86 %). On the other hand, a distinguishable decrease of genetic similarity was observed for all genes' sequences after transformation except *VIM* which showed an increase of genetic similarity as follows: *VIM* (100%), *IMP* (94.73%), *OXA* (96.77%), *NDM* (96.17%), and *KPC* (95.37%). The ten sequences of the wild and transformed genes generated in this study were submitted to GenBank (Accession numbers: LC716395 | 001, LC716396 | 002, LC716397 | 003, LC716398 | 004, LC716399 | 005, LC716400 | 006, LC716401 | 007, LC716402 | 008, LC716403 | 009, LC716404 | 010).

The Correlations between the antibiotic resistance profile of the *K. pneumoniae* isolates, PCR patterns of carbapenem resistant genes, and ERIC genotypes were statistically determined by using the Chi-square test and Monte Carlo method by using the IBM SPSS software package version 20.0 (Armonk, NY: IBM Corp). There was a significant correlation (p ≤ 0.05) between ERIC genotypes and resistance patterns, as well as between resistance patterns and PCR patterns; however, there was no significant correlation (p ≥ 0.05) between ERIC genotypes and PCR patterns.

## 5. Discussion

*K. pneumoniae* is a highly virulent pathogen that can cause pneumonia, liver abscesses, urinary tract infections, and life-threatening septicemia in humans. Carbapenem resistant *K. pneumoniae* is one of the most threatening public health problems worldwide. It has contributed to increasing the mortality rate by as high as 50% constituting a serious threat to public health [25].

In Egypt, previous studies revealed that carbapenem resistance in *K. pneumoniae* isolates is a real problem [26]–[28]. Eighty percent of our collected samples were *K. pneumoniae*, and 75% of them were carbapenem-resistant. In contrast, lower carbapenem resistance percentages were reported by other Egyptian researchers. For instance, Gandor *et al*. and Al Baz *et al*. reported that carbapenem resistance was 66% and 25.4%, respectively among their isolates [27]. Moreover, Indrajith *et al*. and Li *et al*. reported that 58% and 48.1% of their isolates were carbapenem resistant in India and China, respectively [29],[30]. The high percentage of carbapenem resistance in our institution may be due to the extensive use of meropenem as an empiric treatment of infections.

In this study, antimicrobial susceptibility testing for carbapenem-resistant isolates showed multidrug resistance in all isolates. The high resistance to all antibiotic groups is common among carbapenem-resistant isolates. Similarly, Ragheb *et al*. and Yu *et al*. [28],[31] reported multidrug resistance in all carbapenem-resistant isolates in Egypt and China, respectively. This could be attributed to the presence of many resistance genes on plasmids carrying carbapenemases.

In the present study, the VITEK system was used to detect ESBL by the assessment of the inhibitory effects of cefepime, cefotaxime, and ceftazidime, alone and in the presence of the beta-lactamase inhibitor clavulanate [32]. Only 2 isolates (3.3%) of the 60 carbapenem-resistant *Klebsiella* isolates were ESBL-positive. In contrast, in Turkey and Riyadh, Carrer *et al*. and Al Agamy *et al*. reported that 94.9% and 90.5% of their carbapenem resistant isolates were ESBL positive, respectively [33],[34].

Epidemiological studies of carbapenem resistance and investigating different types of carbapenemases in hospital settings have a great influence on infection control and treatment [35]. Molecular identification of carbapenemases has the advantage of the ability to detect low-level resistance carbapenemase producers, which are difficult to be detected by conventional techniques [36]. In this study, molecular detection of carbapenem resistance genes using conventional PCR technique showed that all isolates contained at least two carbapenem resistance genes. All isolates were positive for the *OXA* gene, 56 (93%) were positive for the *VIM* gene, 51 (85%) were positive for the *NDM* gene, 38 (63%) were positive for the *IMP* gene, and 10 (17%) were positive for *KPC* gene. In Egypt, the *OXA* gene was reported as the most predominant carbapenemase with no or low prevalence of the *KPC* gene in many studies between 2020 and 2022 [37]–[39]. In contrast, Gandor *et al*. in 2022 reported that the *NDM* gene was the most prevalent gene followed by *OXA* and *KPC* genes [27]. Meanwhile, many studies between the years 2015-2018 in Egypt reported the *KPC* gene as the most predominant gene [40],[41]. On the other hand, Davarci *et al*. reported that the most prevalent carbapenemase gene in Turkey was *OXA* followed by *NDM* and *VIM* genes; however, *KPC* and *IMP* genes were not detected in any of the tested isolates [42]. In contrast, Tsilipounidaki *et al*. reported that the most prevalent carbapenemase gene was *KPC*, followed by *VIM* and *NDM* in Greece [43]. In Iran, Alizadeh *et al*. reported that the *OXA* gene was the most prevalent carbapenemase among their isolates, followed by *NDM*, *IMP*, *VIM*, and *KPC* [44].

In this study, molecular detection of carbapenemases genes revealed that all isolates contained at least two of the carbapenem resistance genes. Seven (12%) of the 60 *K. pneumoniae* isolates were positive for five resistance genes, 26 (43%) were positive for four genes, 22 (37%) were positive for 3 genes, and 5 (8%) were positive for two genes. The coexistence of more than one carbapenemase augmented the carbapenem hydrolyzing effect especially since the weak *OXA-48* gene did not exist alone in any isolate. The co-production of various types of resistant genes could be alarming and requires more investigation. Similarly, Raheel *et al*. detected a combination of 2 or more carbapenemase genes in 90.6% of their isolates. The largest number of isolates (45.3%) carried the 3 genes, *VIM*, *OXA* and *NDM* [38]. In contrast, Gandor *et al*. reported 21% of their samples contained more than 1 carbapenemase; 41% of them contained 3 genes [27]. On the other hand, Tsilipounidaki *et al*. detected a combination of 2 carbapenemases in 25% of their carbapenem-resistant isolates in Greece [43].

Since *K. pneumoniae* plays an important role in nosocomial infections, genotyping of clinical isolates is useful for identifying infection sources and thus preventing hospital-acquired infections. In this study, the ERIC-PCR technique was used for the molecular typing of our clinical isolates because it is a quick, reliable, and cost-effective technique [45]. Dendrogram analysis of *K. pneumoniae* isolates showed 57 individual patterns, arranged in three clusters showing the genetic diversity among our isolates. Genetic diversity of clinical *K. pneumoniae* isolates were reported in other studies reflecting high heterogenicity. Similarly, Mehr *et al*. reported that their 35 clinical *K. pneumoniae* isolates demonstrated 32 ERIC profiles in Iran [23]. Moreover, Mohamed *et al*. reported that 23 carbapenem-resistant isolates harbored 17 pulsotypes in Egypt [46].

In this study, there was a significant correlation between ERIC genotypes and resistance patterns, and between resistance patterns and PCR patterns; however, there was no significant correlation between ERIC genotypes and PCR patterns. This result demonstrated that plasmid-mediated carbapenemases are not restricted to specific clones because the ERIC-PCR amplified repetitive intergenic regions of the bacterial chromosomes only, not plasmid regions which contained most of the resistance genes. Similar to our results, a significant correlation between *K. pneumoniae* ERIC profiles and sensitivity patterns was also reported by Wasfi *et al*. in Egypt [18]. In addition, Sedighi *et al*. and Oliveira *et al*. reported the absence of a significant correlation between *K. pneumoniae* genotypes and PCR patterns of plasmid-mediated resistance genes in Iran and Brazil [47],[48].

In this study, the five carbapenemases were successfully transformed from resistant *K. pneumoniae* isolates to sensitive *K. pneumoniae* and *E. coli* isolates reflecting the high risk of horizontal resistance transfer. The transformation of the five genes increased the MIC of imipenem in all isolates (*E. coli* and *K. pneumonia*e). In contrast, Ragheb *et al*. reported that only the *VIM* gene was successfully transferred in Egypt [28]. In addition, Sun *et al*. reported the successful transfer of *KPC* and *IMP* genes from 2 of 9 isolates of *K. pneumoniae* to *E. coli* EC600 in China [49].

In this study, ERIC-PCR was also employed separately to detect genetic diversity before and after transformation for four *K. pneumoniae* isolates; a phylogenetic tree was constructed showing high genetic similarity among the wild strains which were arranged in the same cluster, and among the transformed isolates which were arranged in another cluster. Comparing *IMP*, *VIM, OXA, NDM*, and *KPC* gene sequences before and after bacterial transformation reflected distinguishable differences. Genetic sequences of wild isolates were more similar to reference genes in the Genebank than genetic sequences of transformed isolates indicating the superior influence of the transformation process on bacterial genome.

In conclusion, plasmid-mediated carbapenem resistance in *K. pneumoniae* and its dissemination among different strains is a real threat to public health. ERIC-PCR is a very effective tool for detecting the genetic diversity among strains. To the best of our knowledge, the high prevalence of carbapenemase genes hasn't been detected in any previous Egyptian study.
